# The Bimodality Index: A Criterion for Discovering and Ranking Bimodal Signatures from Cancer Gene Expression Profiling Data

**DOI:** 10.4137/cin.s2846

**Published:** 2009-08-05

**Authors:** Jing Wang, Sijin Wen, W. Fraser Symmans, Lajos Pusztai, Kevin R. Coombes

**Affiliations:** 1 Department of Bioinformatics and Computational Biology, The University of Texas M.D. Anderson Cancer Center, 1515 Holcombe Blvd., Houston, TX 77030-4009; 2 Department of Biostatistics, The University of Texas M.D. Anderson Cancer Center, 1515 Holcombe Blvd., Houston, TX 77030-4009; 3 Department of Pathology, The University of Texas M.D. Anderson Cancer Center, 1515 Holcombe Blvd., Houston, TX 77030-4009; 4 Department of Breast Medical Oncology, The University of Texas M.D. Anderson Cancer Center, 1515 Holcombe Blvd., Houston, TX 77030-4009. Email: kcoombes@mdanderson.org or jingwang@mdanderson.org

## Abstract

**Motivation:**

Identifying genes with bimodal expression patterns from large-scale expression profiling data is an important analytical task. Model-based clustering is popular for this purpose. That technique commonly uses the Bayesian information criterion (BIC) for model selection. In practice, however, BIC appears to be overly sensitive and may lead to the identification of bimodally expressed genes that are unreliable or not clinically useful. We propose using a novel criterion, the bimodality index, not only to identify but also to rank meaningful and reliable bimodal patterns. The bimodality index can be computed using either a mixture model-based algorithm or Markov chain Monte Carlo techniques.

**Results:**

We carried out simulation studies and applied the method to real data from a cancer gene expression profiling study. Our findings suggest that BIC behaves like a lax cutoff based on the bimodality index, and that the bimodality index provides an objective measure to identify and rank meaningful and reliable bimodal patterns from large-scale gene expression datasets. R code to compute the bimodality index is included in the ClassDiscovery package of the Object-Oriented Microarray and Proteomic Analysis (OOMPA) suite available at the web site http;//bioinformatics.mdanderson.org/Software/OOMPA.

## Introduction

Identifying genes with bimodal expression patterns from large-scale expression profiling data is an important task. Bimodal expression patterns can result naturally from differential expression, with the two modes centered on the mean expression of a gene in two distinct subgroups of samples. In the context of cancer, bimodal expression patterns can result from genomic lesions that occur in some patients but not others. For example, Tomlins and colleagues^1^ noticed that the ETV1 gene was overexpressed in 10%–20% of prostate cancer samples in multiple data sets and that the ERG gene was overexpressed in about 40% of prostate cancer samples in the same datasets. They further noticed that overexpression of these two genes was mutually exclusive. These findings led them to discover that the overexpression was driven by recurrent translocations that fused the androgen-responsive gene TMPRSS2 either with ETV1 (in some cases) or with ERG (in other cases).

The definition of a bimodal distribution can be vague: the term typically refers to a mixture of two populations with distinct means. In density estimation, a bimodal distribution can be recognized by the presence of two modes, each with a characteristic peak. Determining the factor that characterizes the samples that belong to each of the two distributions can be difficult. In the realm of large-scale gene expression profiling, finding bimodal expression patterns is an important analytical process. In oncology, this process may be part of the search for clinically important therapeutic targets within tumors. The process can also reveal molecular signatures that distinguish tumor subtypes, which contributes to our understanding of the clinical and biologic characteristics of cancer.

A two-component normal mixture-model-based clustering algorithm is commonly used to discover bimodal expression patterns. In contrast to other clustering methods, this approach is based on a mixture of statistical distributions in which each component represents a cluster. The method converts a classification problem into a statistical estimation of the mixture density. The approach has proven to be useful in a wide range of applications, including microarray gene expression analysis.^2^–^6^ The technique is sensitive and allows for rapid computation. A major benefit of the mixture model-based clustering technique is that the algorithm characterizes each cluster and provides probabilities of cluster membership. In addition, the algorithm provides point estimates of the statistical parameters including the means, standard deviations, and sample proportions in each group.

One difficulty with its application is finding an appropriate test statistic to estimate p-values and choosing a suitable cutoff to minimize the false discovery rate. The log likelihood ratio test (LRT) statistic could be adopted to test the hypothesis H_1_ that gene expression distribution is a bimodal, against the null hypothesis H_0_ that the distribution is unimodal. Under the null hypothesis, the LRT asymptotically has a chi-squared distribution (−2 log λ ~ χ^2^). However, in practice, the chi-squared distribution, with two degrees of freedom, seems to converge slowly. Consequently, the critical value from chi-square table is too small, so it over-rejects, or inflates the false positive rate.^7^ Recently Ertel and Tozeren applied a two-component normal mixture model to identify bimodal genes and their potential roles in cell signaling and disease progression.^8^ They used LRT, with p-values estimated by evaluating the chi-square distribution with six degree of freedom, in order to get more conservative p-values. To identify significant bimodal genes, an ad-hoc p-value was selected.

Researchers combine mixture-model-based clustering with either the Akaike information criterion (AIC) or the Bayesian information criterion (BIC). Both criteria impose arbitrary penalties based on the number of parameters to determine whether a unimodal or bimodal model is a better fit to the observed data. A dataset is identified as bimodal if the parameter penalties are outweighed by the increased likelihood of the bimodal model. Although useful, according to our experience, the reliance on AIC or BIC appears to be overly sensitive in its application to gene expression profiling data and may lead to the identification of numerous genes whose bimodal patterns cannot be confirmed in follow-up studies. In order to be clinically useful in practice, a bimodal pattern should exhibit significant separation between the means of the two groups and should have adequate sample sizes in each group. In applications of the mixture-model-based clustering technique, researchers frequently find it necessary to use subjective ad hoc cutoffs in addition to the AIC or BIC in order to reveal reliable and meaningful bimodal patterns.

For identifying bimodal expressed genes, a practical method was introduced previously by Andrew and colleagues,^9^ called Profile Analysis using Clustering and Kurtosis (PACK). PACK has two steps; (1) using the expectation-maximization (EM) algorithm, a common method for finding maximum likelihood estimates of parameters in probabilistic models, and BIC for model selection to determine the number of clusters within the expression data. This step is the same as the model-based-clustering technique. (2) Using kurtosis to characterize features and find relevant classifiers. Depending on the sign of kurtosis, major bimodal pattern (negative kurtosis) or outlier bimodal pattern (positive kurtosis) can be identified. PACK has been applied to number of cancer gene expression datasets in breast successfully.^9^,^10^ The use of kurtosis for identifying bimodally expressed genes, however, has a blind spot. If the proportions of samples in the two groups are close to a 20%–80% split, then the kurtosis is close to zero. Negative kurtosis strongly favors a perfect 50–50 split, which we think has a chance to miss biologically interesting genes. Positive kurtosis, on the other hand, shows a strong preference for extremely unbalanced splits on the order of 95–5 or even 99–1.

In this investigation, we propose an approach that replaces (or supplements) the use of AIC or BIC with a criterion that provides for finer distinctions between the bimodally expressed genes. By assigning a continuous value (the bimodality index) to each gene instead of a simple yes-or-no answer to the question of bimodality, we can provide researchers with a tool to rank genes and thus focus their interest on those with the strongest evidence of “useful” bimodality. We evaluate the estimation of the bimodality index of a set of measurements using both mixture model-based clustering and Markov chain Monte Carlo (MCMC) techniques to estimate the statistical parameters (means, standard deviation, and sample proportions) of the mixture. MCMC sampling techniques are common in the field of Bayesian analysis, and have emerged as popular tools for the analysis of complex statistical problems, including the analysis of microarray gene expression profiling data.^11^,^12^ We apply the proposed algorithm to simulated gene expression data and to a dataset from a microarray gene expression profiling study of breast cancer.

## The Algorithm in Brief

We assume that, for a gene with bimodal expression, the distribution can be expressed as a mixture of two normal distributions:

(1) $$y=\pi N(\mu_{1},\sigma_{1})+(1-\pi )N(\mu_{2},\sigma_{2})$$

where *y* is the expression measurement; *π* is the proportion of samples in one group; *μ*_1_ and *μ*_2_ are the means of the expression level of the two modes; and *σ*_1_ and *σ*_2_ are the standard deviations. In analyzing gene expression data, equal variance between groups is frequently assumed. If we assume that *σ*_1_ = *σ*_2_, the equation becomes

(2) $$y=\pi N(\mu_{1},\sigma )+(1-\pi )N(\mu_{2},\sigma )$$

where *σ* is the common standard deviation. We define the standardized distance, *δ*, between the two populations as

(3) $$\delta =\frac{\left|\mu_{1}-\mu_{2}\right|}{\sigma }.$$

For identifying genes with bimodal expression, the null hypothesis is *δ* = 0 and the alternative hypothesis is *δ* > 0.

**Figure 1:**
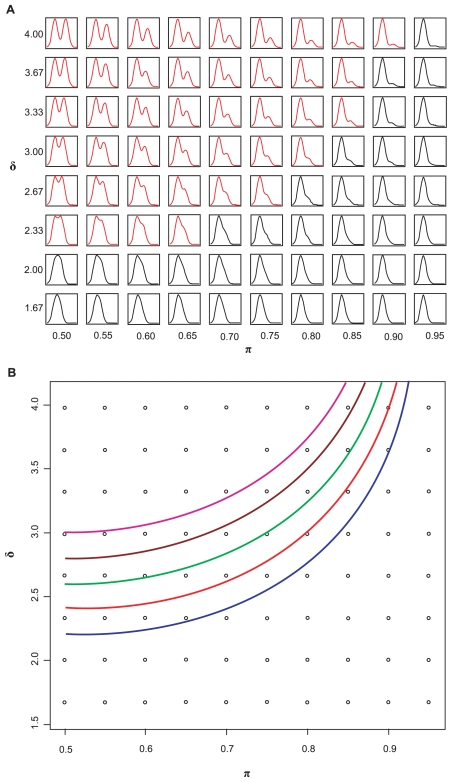
Relationships between bimodality and *π* and *δ*. **(A)** Density plots of bimodal data as *π* and *δ* vary. These plots indicate that bimodality is obvious when both *π* and *δ* are sufficiently large, but difficult to distinguish when *π* and *δ* reach certain values. The density plots colored red are “visually” distinguishable as bimodal measurements. **(B)** Set of quadratic curves computed using different *BI* values; *BI* = 1.1 (blue), 1.2 (red), 1.3 (green), 1.4 (brown), and 1.5 (purple).

To illustrate how the shape of a bimodal density changes as *π* and *δ* vary (with *σ* = 1), we plotted a set of theoretical distributions. Figure 1a arranges the density plots in the *δ – π* plane. Because of symmetry in *π* from 0.0 to 1.0, we only illustrate the plots using *π* from 0.50–0.95. The plots indicate that bimodality is visually obvious when *δ* is sufficiently large or when the proportion *π* in each group is adequate. When *π* and *δ* reach certain critical values, bimodality is no longer visually distinguishable. The plots also suggest that the distinguishable bimodal patterns, as indicated by the plots in red in Figure 1a, are approximately bounded by a curve in the *δ – π* plane. Although this observation about distinguishability is subjective, it can be made objective by reference to a standard sample size computation.

### Defining the bimodality index

Consider an experiment that involves two normally distributed populations with means *μ*_1_ and *μ*_2_ and common standard deviation *σ*. The usual sample size computation tells us that the formula

(4) $$N=\frac{4{\left(Z_{\alpha /2}+Z_{\beta }\right)}^{2}}{\delta^{2}},$$

where *z_α/_*_2_ and *z_β_* are the percentiles of the standard normal distribution that yield the desired significance and power, gives the total number *N* of samples needed to detect the standardized difference *δ* in an experiment with equal-sized groups. To achieve the same power with unequal sizes *Mπ* and *M*(1 – *π*), we should choose *M* to make the variance of the estimated standardized difference with unequal group sizes the same as the variance with equal sizes; that is,

(5) $$\frac{1}{\pi (1-\pi )M}=\frac{1}{\pi M}+\frac{1}{(1-\pi )M}=\frac{1}{N/2}+\frac{1}{N/2}=\frac{4}{N}.$$

So, the total number of samples required when the groups are unequally sized is:

(6) $$M=\frac{{\left(Z_{\alpha /2}+Z_{\beta }\right)}^{2}}{\pi (1-\pi )\delta^{2}}.$$

Selecting reasonable values for *α* and *β* for microarray experiments has been addressed by Simon et al,^13^ so we will not provide a detailed description here. Rearranging equation (6), we obtain

(7) $$\pi (1-\pi )\delta^{2}=\frac{{\left(Z_{\alpha /2}+Z_{\beta }\right)}^{2}}{M}.$$

Motivated by equation (7), we define the bimodality index *BI* as

(8) $$BI={\left[\pi (1-\pi )\right]}^{1/2}\delta .$$

In practice, we can estimate *δ* and *π* for a given dataset, then use these estimated values to compute *BI*.

Combinations of *δ* and *π* that give the same value of *BI* describe bimodal distributions that are “equally separable” in the sense that experiments to distinguish the two subgroups at a given significance and power would require the same total number of samples. The right hand side of equation (7) shows that larger values of *BI* correspond to smaller sample sizes and thus represent bimodal distributions that are easier to distinguish. Constant *BI* values in equation (8) define curves in the *δ – π* space (Fig. 1b). Because of symmetry in *π* (0.0–1.0), we only show the plot for *π* from 0.5–1.0. The curves with constant bimodality index take on their minimum value at *π* = 0.5 (when the sizes of the two subgroups are the same), which gives the most power to distinguish a bimodal pattern for a given total sample size. When the group sizes are very unequal, for example, when *π* is close to 0.1 or 0.9, the power is much weaker for the same total sample size. Because the curve defined by *BI* = 1.1 perfectly separates the red “visually bimodal” distribution curves from the black distribution curves in Figure 1a, we recommend this cutoff to select bimodally expressed genes. Other cutoffs can be chosen by using equations (7) and (8) to compute the sample size that would be needed to validate this degree of bimodality in an independent data set.

The key issue that remains, then, is how to accurately estimate the parameters *δ* and *π* (and thus *BI*) from a given dataset. One approach is to use the expectation-maximization (EM) method for maximum-likelihood estimation in parameterized normal mixture models. We can then combine the EM method for mixture estimation with BIC for identifying bimodal distributions. We can use the R package MCLUST^©^ (University of Washington, Seattle, Washington), which follows this approach. We refer to this approach as a normal mixture-model-based clustering algorithm. Alternatively, we can use MCMC sampling techniques for the estimation in a parameterized normal mixture model. We applied both algorithms to estimate the statistical parameters of the measurements. Then, based on the estimated parameters obtained from both algorithms, we computed *BI* in order to identify and rank more reliable bimodal patterns of expression. The difference between the two algorithms is their output: the mixture model-based clustering technique provides point estimates, whereas the MCMC technique provides distributions of the estimated parameters. Knowing the distributions of the estimated parameters allows us to estimate the posterior probability of the classification of each sample.

## Simulation Studies

To evaluate the performance of the bimodality index for identifying genes with meaningful bimodal expression, we first performed simulation studies. We adopted the R package MCLUST^©^ (University of Washington, Seattle, Washington), to perform mixture-model-based clustering and to obtain the estimated statistical parameters. From these estimated values, we computed the bimodality index *BI* for each measurement, and then selected bimodal measurements by setting a cutoff on the *BI* value. For comparison, we applied a hybrid MCMC technique to the same simulated dataset to estimate the measurement parameters and compute *BI*. To compute *BI*, we used point-estimated parameters from MCLUST and the posterior mean estimates from MCMC.

### Unimodally distributed measurements

To obtain unimodal distributions, we generated expression datasets at four sample sizes: *n* = 50, 100, 200, and 300. Each simulated dataset consisted of 500 randomly generated samples from a unimodal normal distribution, *N*(*μ*= 5, *σ* = 1). Such a study is used to evaluate the rate of false positives or to evaluate the specificity of the proposed method.

The results of our null simulation are presented in Table 1. When the sample size was sufficiently large (≥200), our proposed method using estimated parameters from either MCLUST or MCMC performed equally well, with almost no false discoveries. Similarly, the rate of false positives was low when using BIC. When the sample size was relatively small (*n* < 100), the MCMC algorithm provided slightly better results. All three methods performed with high specificity in the simulated null datasets, which indicates very low rates of false positives.

### Bimodally distributed measurements

Next, we evaluated the proposed method on simulated measurements that were truly bimodally distributed. Here the simulations are more elaborate, since *δ* and *π* will affect the “strength” or “reliability” of the bimodal expression. To fully evaluate the performance of the proposed method, we simulated expression datasets with different parameter settings.

#### Various *δ* with sufficient *π* in each group (*π* = 0.3–0.7)

To evaluate how the proposed method detected bimodal measurements for different values of *δ*, we simulated datasets as *δ* varied from 2 to 5. Here, *δ* ≥ 4 indicates a strong bimodal pattern; *δ* = 3 indicates a weak bimodal pattern; and *δ* = 2 corresponds to a very weak bimodal pattern. We assumed equal variance in each group. For simplicity, we set *σ*_1_ = *σ*_2_ = 1, in which case *δ* is equivalent to Δ*μ*. We again used four sample sizes, *n* = 50, 100, 200, and 300. For this simulation, we let *π* range from 0.3 to 0.7 by steps of 0.1. For each *π*, we simulated 100 bimodal measurements. Therefore, we had 500 bimodal variables associated with different values of *π* for each sample size. We applied both MCLUST and MCMC to estimate the statistical parameters *μ*_1_, *μ*_2_, *σ*, *π*, and *δ*. Based on these estimated parameters, we computed *BI* for each measurement.

**Figure 2:**
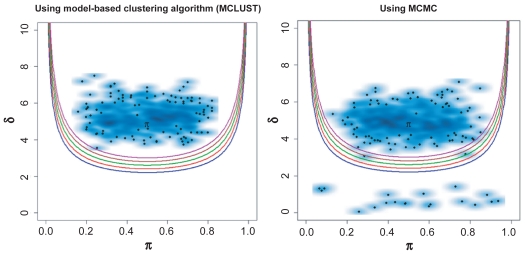
Performance of the proposed method with MCLUST and MCMC techniques in a simulated strong bimodal measurement dataset, *δ* = 5 and *π* = 0.3–0.7. The dataset contains 50 samples and 500 measurements. The black spots represent individual measurement, and blue clouds indicate the density of each data point. The set of quadratic curves computed using different *BI* values; *BI* = 1.1 (blue), 1.2 (red), 1.3 (green), 1.4 (brown), and 1.5 (purple).

The results from this simulation study indicate that for a large *δ* (*δ* ≥ 4), the percentage of identified bimodal measurements using MCLUST was close to the percentage obtained using MCMC. Both techniques demonstrated high accuracy (Table 2a). When *δ* = 5, the MCLUST algorithm facilitated the identification of the bimodal measurements with no false negatives (Fig. 2). This was also true when BIC was used. Even with a small sample size (*n* = 50) we were able to identify nearly all of the samples as bimodal. As expected, when the separation of the means between the two groups was large and the sample population in each group was sufficient (30% ≤ *π* ≤ 70%), any of the three methods allowed us to easily identify the bimodal measurements. For *δ* = 3, the identification of bimodal measurements was the same using any of the three methods. When the sample size was small (*n* ≤ 50), all three methods identified about 50% of the bimodal measurements. As the sample size increased, all three methods performed equally well and identified nearly 100% of the bimodal measurements.

**Figure 3:**
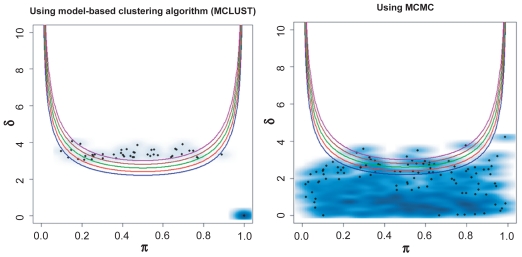
Performance of the proposed method with MCLUST and MCMC in simulated bimodal dataset, with *δ* = 2 and *π* = 0.3–0.7. The dataset contains 50 samples and 500 measurements. The set of quadratic curves computed using different *BI* values; *BI* = 1.1 (blue), 1.2 (red), 1.3 (green), 1.4 (brown), and 1.5 (purple).

When *δ* = 2, which indicates a very weak bimodal pattern and is slightly below our defined detection limit (bimodality index, *BI* = 1.1), we expect that most of the measurements will be identified as unimodal. Our study showed that only a few measurements were identified as bimodal under this setting (Fig. 3 and Table 2a). The results suggest that the MCMC method is slightly less sensitive than the MCLUST algorithm, but that the difference is almost negligible. Table 1 also shows that the BIC is more sensitive, especially with a larger sample size. This is not surprising. Because *δ* = 2, these simulations do arise from bimodal distributions. In effect, the BIC behaves like a cutoff on the bimodality index at a much smaller value than our limit of *BI* = 1.1 and thus will identify more samples as bimodal.

#### Various *δ* with small proportions in one group (*π* = 0.1, 0.2, 0.25, or 0.8, 0.9)

**Figure 4:**
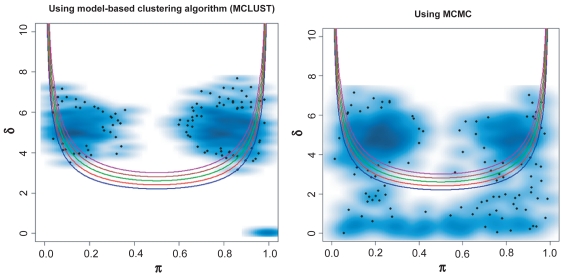
Performance of the proposed method with MCLUST and MCMC techniques in a simulated bimodal dataset, with *δ* = 4 and *π* = 0.1, 0.2, 0.25, 0.8 and 0.9. The simulated dataset contains 50 samples and 500 measurements. The set of quadratic curves computed using different *BI* values; *BI* = 1.1 (blue), 1.2 (red), 1.3 (green), 1.4 (brown), and 1.5 (purple).

We then focused on the issue of highly unbalanced group sizes (summarized in Table 2b). The findings indicate that the bimodality index performed similarly when using either MCLUST or MCMC in settings with a strong bimodal pattern, i.e. when *δ* was large (*δ* ≥ 4) and the sample size was sufficient (*n* ≥ 100). Using MCMC, the performance was consistently more conservative. The BIC method was associated with a higher percentage of identified bimodal measurements, regardless of the sample size. The study also indicated that, when *π* was less than 0.1 or greater than 0.9 in one group, some bimodal measurements were detected as unimodal (Fig. 4). In other words, when *π* ≤ 0.1 or *π* ≥ 0.9 and the sample size is small (*n* ≤ 100), even when *δ* is sufficiently large, the false-negative rate will be high.

## Application to Cancer Microarray Gene Expression Measurements

Microarray gene expression measurements are more complicated than simulated data. To evaluate the usefulness of our proposed method for identifying genes with bimodal expression, we applied the algorithm to a dataset of microarray gene expression profiles from a study of breast cancer.

### Briefs of expression profiling and data processing

The gene expression profiling array dataset was produced by the Breast Cancer Pharmacogenomic Program at The University of Texas M. D. Anderson Cancer Center using Affemetrix U133A GeneChip. The dataset contains 133 human breast cancer samples, with each array containing 22,283 probe sets. (The expression profiles and clinical information are available at http://bioinformatics.mdanderson.org/pubdata.html.) The original purpose of the investigation was to develop multi-gene predictors of pathologic complete response (pCR) to preoperative therapy.^14^ The clinical variables associated with the dataset include disease stage, histologic grade, and routine clinical markers such as the estrogen receptor (ER), the human epidermal growth factor receptor 2 (HER-2), and the progesterone receptor (PR) status. Response to preoperative chemotherapy was also available.

For gene expression measurements, the signal intensities at the level of the probe sets were quantified by dChip 1.3 (http://dchip.org) using the PM-model only. Normalization was performed by dChip using the array with median brightness. The normalized expression measurement was logarithm transformed (base 2) for analysis. Because the expression data were produced on different dates, we considered the possibility of a “batch effect,” which occurs as a result of differences in the hybridization environment on different days. Although common in microarray gene expression profiling investigations, the batch effect necessitates correction of the data. In order to obtain meaningful results, we performed a gene-by-gene adjustment of the means to put the batches created on two different dates on the same scale. Briefly, we adjusted the means on each gene from one batch to match the means of the same genes in the other batch.

### Identifying genes with bimodal expression

**Figure 5:**
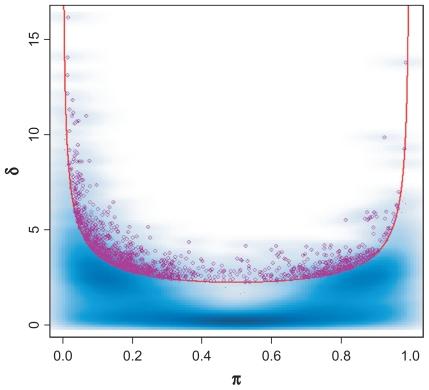
Genes identified with bimodal expression from breast cancer dataset. The significant genes with bimodal expression are circled in purple. The *π* represent the population sizes; *δ* is the difference between the means divided by standard deviation (*σ*). Equal variance in both groups is assumed. The curve represents *BI* = 1.1.

Once data processing was complete, we applied the proposed method with the MCLUST and MCMC algorithms to identify the genes with bimodal expression. In both approaches, we estimated *μ*_1_, *μ*_2_, *σ*, *π*, and *δ* from the expression measurement of each gene across all samples, and computed *BI* for each gene. Figure 5 shows the genes from the breast cancer dataset identified as having bimodal expression.

Microarray gene expression profiling data contain significant levels of technical and biologic variation. Many measurements are just “noise,” containing no useful information. In order to identify genes with interesting expression patterns, it is common to apply a filtering criterion to eliminate some “noise.” The selection of a filtering condition involves some subjectivity. In this investigation, we filtered at several different levels. Specifically, we first computed the expression levels at several percentiles (0.25, 0.30, 0.35, 0.40, 0.45, and 0.50) using all expression measurements across all samples. We then computed the maximum expression value of each gene across all samples. If the maximum was less than the expression level at a selected filtering percentile, we considered that expression measurement to be “noise” and eliminated it from the analysis. For example, the overall 25th percentile of expression was 6.771. If a gene had a maximum expression of less than 6.771, we removed that gene from the analysis. Once the “noise” measurements were filtered out, we performed the analysis for identifying bimodal measurements using the MCLUST and MCMC techniques at each of the defined filtering percentiles and at each of the *BI* values. The results are summarized in Table 3.

The results show that using our method combined with MCLUST identified more genes with bimodal expression than using our method combined with MCMC at the same filtering conditions and the same bimodality index cutoff. This suggests again that MCLUST is more sensitive than MCMC. However, at every level of the bimodality index and at every filtration level based on a more stringent definition of noise, a larger percentage of the genes identified by MCMC remain above the noise, as compared to MCLUST. We also compared the genes with bimodal expression that were identified from our method combined with the MCMC versus the MCLUST algorithms. The results from that comparison show extensive overlap in the genes identified with bimodal expression by both algorithms (see Table 4). This suggests that using our method with either technique yields similar results when estimating the statistical parameters and computing the bimodality index for detecting genes with bimodal expression.

**Figure 6:**
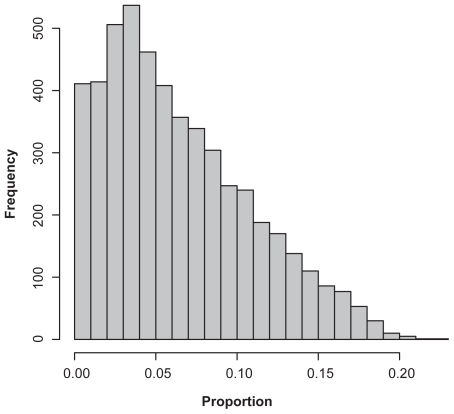
Histogram of the proportion of samples in the smaller of two groups for genes that were called bimodal by BIC in the breast cancer dataset but had BI < 1.1.

The results also show that vastly more genes (often by an order of magnitude) were identified as bimodal using BIC, but a smaller percentage of these genes remain above the filtration noise cutoffs. Because the bimodality index for these genes is small, this finding strongly suggests that the majority of the genes identified by BIC either have small separations between the modes or have highly imbalanced proportions of samples in the two groups. Figure 6 illustrates that 70% of genes called bimodal by BIC that have *BI* < 1.1 also have *π* < 10%, and 97% of these genes have *π* < 20%. Thus, most of these genes are driven by a relatively small number of samples in one of the two groups.

### Example of genes identified as having bimodal expression in the human breast cancer data

With known clinical information from the dataset, we checked three probesets related to three breast cancer genes, ERS1, PGR and HER2, which we expect to be expressed bimodally in our dataset. Previous investigation suggested that the three selected probesets correspond to the three genes strongly.^15^–^17^ In addition to the three known genes in breast cancer, we also present here two bimodal genes (CKB and BST2) discovered by our method.

**Figure 7:**
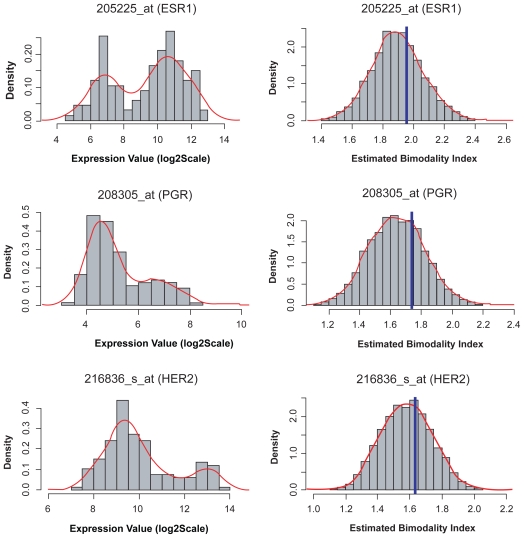
**Left panel**: histograms of three identified bimodally expressed genes from breast cancer dataset; estrogen receptor (top), progesterone receptor (middle), and HER-2 (bottom). **Right panel**: posterior distributions of bimodality index on the three genes, computed using MCMC. The vertical blue lines indicate the point estimated bimodality index value from MCLUST. The red lines are the density estimations.

The first example is the estrogen receptor (ERS1) with probe set ID 205225_at. From a previous analysis, we know that this probe set correlates highly with the clinical estrogen receptor status.^16^ Figure 7 illustrates the density plot for the ERS1 gene (top left). The estimated parameters are provided in Table 5. The point estimate is *BI* = 1.955 and the posterior probability of being bimodal is > 99.9% (based on *BI* = 1.1). This value of *BI* was the 16th largest among the 22,283 probe sets on the array. The clinical information associated with this experiment indicates that there are 51 patient samples with negative ER status and 82 patient samples with positive ER status in this dataset. The estimated proportions are close to the clinical ER status obtained from both the MCMC and MCLUST techniques. In comparison, MCLUST provided slightly better results.

The second example of a gene with bimodal expression involves the progesterone receptor (PGR), for which the probe set ID is 208305_at. PGR is an intercellular steroid receptor that specifically binds progesterone and is located at 11q22. The point estimate is *BI* = 1.733, and the posterior probability of it being bimodal is 100%. The density plot of PGR is illustrated in Figure 7 (middle left). The estimated measurement parameters are provided in Table 4. The clinical information indicates that the dataset contains information on 55 patient samples with positive PGR status, 75 patient samples with negative PGR status, and 3 patient samples with unknown PGR status. The estimated proportions in each group are close to those of the true sub-population (see Table 5).

The third example of bimodal gene expression involves the human epidermal growth factor receptor 2 (ERBB2 or HER2), for which the probe set ID is 216836_s_at.^15^,^17^ HER2 is important for its role in the pathogenesis of breast cancer and as a current target of treatment. The density plot of HER2 is illustrated in Figure 7 (bottom left). The estimated measurement parameters are provided in Table 5. The point estimate is *BI* = 1.634 and the posterior probability of HER2 being bimodal is >99.9% (at *BI* = 1.1). The clinical information indicates that the dataset includes information on 33 patient samples with positive HER2 tumor status, 99 patient samples with negative HER2 status, and one patient sample with unknown HER2 status. The estimated proportions in each group approximate those of the true sub-population. The results from these three examples strongly indicate that the proposed method works accurately with the MCMC and MCLUST algorithms. Importantly, all three of these bimodally expressed genes represent therapeutic targets for anti-estrogen and anti-HER2 therapies that are currently used in the clinic. We hope that the other bimodally expressed genes contain similarly important but novel drug targets.

**Figure 8:**
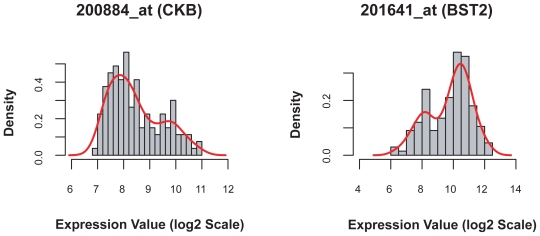
Histograms of two bimodally expressed genes in breast cancer: Creatine kinase, brain (left), and Bone marrow stromal cell antigen 2 (right). The roles of these two genes in breast cancer chemotherapeutic treatment were not reported previously.

In addition to the three “standard” breast cancer therapeutic target genes, we present two bimodal genes identified by our method, but previously unreported. The two genes are (1) Creatine kinase, brain (CKB; with probe set ID 200884_at), and (2) Bone marrow stromal cell antigen 2 (BST2; with probe set ID 201641_at). Both genes exhibit strong bimodal patters, with bimodality index 1.619 for CKB and 1.602 for BST2 (Fig. 8). Their roles in breast cancer are poorly understood and further investigation will be carried out.

### Identifying genes with strong bimodal expression

**Figure 9:**
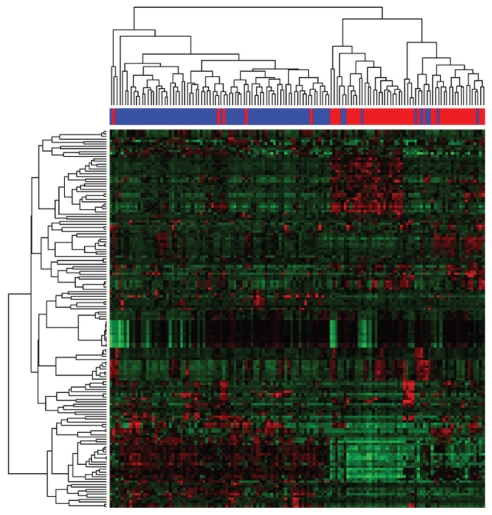
Heat map image produced using 151 genes with strong bimodal expression as commonly identified by MCMC and MCLUST from 133-array breast cancer dataset. Two distinct sample clusters can be seen. One cluster contains mainly ER positive tumor samples (blue color bar) and the other cluster contains mostly ER negative tumor samples (red color bar).

**Figure 10:**
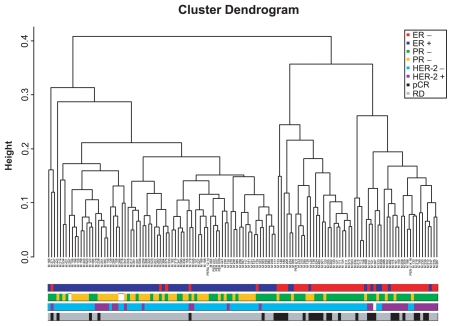
Hierarchical cluster analysis using 151 genes with “strong” bimodal expression as commonly identified by MCMC and MCLUST algorithms. The color codes correlate the ER, HER-2, PR and pCR, and RD status of the tumor.

We also applied more stringent conditions to identify genes with bimodal expression. Using *BI* ≥ 1.5 as a cutoff and without filtering, we identified 181 and 213 genes with bimodal expression using the MCMC and MCLUST algorithms, respectively. There were 151 genes with bimodal expression that were identified using both algorithms. To test the hypothesis that bimodally expressed genes could be used as outcome or disease phenotype markers, we performed two-way hierarchical cluster analysis using these 151 genes (Fig. 9). The results of the analysis suggest that the genes with the strongest bimodal expression are closely related to two clinical types of breast cancers: ER-positive cancers and ER-negative cancers. As these two neoplastic diseases of the breast may originate from different cell types (luminal and basal epithelial cells, respectively) this association is not surprising and supports the validity of our method.^18^ We also correlated the clusters with HER2 status and with patient response to treatment, recorded as either pCR or residual disease (RD) (see Fig. 10). As noted, the major split in the dendrogram correlates with hormone receptor status. The second split in the dendrogram correlates with HER-2 status. These associations have already been noted in the clinical literature.^17^ However, correlations between these routine clinical markers and the other genes on our list will warrant further biological exploration.

## Discussion and Conclusions

In this paper, we have introduced a new approach to identifying and ranking meaningful and reliable bimodal measurements from large-scale gene expression measurements. The key to this approach is to define a bimodality index that provides an objective measure of the “strength” of the bimodal separation. Comparing with AIC or BIC, the major difference is that our approach is not just for selecting bimodal genes, but also ranks the genes. The bimodality index can be evaluated empirically, and its interpretation is justified by standard sample size and power calculations. To apply this method to large-scale gene expression data, we need to estimate the model parameters *μ*_1_, *μ*_2_, *σ*, and *π* in order to compute the bimodality index *BI*. Suitable techniques for performing such an analysis include a mixture-model-based clustering technique and the MCMC technique.

When applying a mixture-model-based clustering technique, the number of components (clusters) needs to be predefined. For bimodal measurements, a two-component mixture model is applied. The model fits the data with a mixture of two normal distributions, and provides point estimations of the measurement parameters *μ*_1_, *μ*_2_, *σ*, and *π*. The estimated parameters are then used to compute the bimodality index. The major advantage of the mixture-model-based clustering technique is computational efficiency. For large-scale gene expression data, that process can be completed in a very short time with high accuracy in the estimated parameters.

The advantage of the MCMC technique is that it provides distributions of the parameters *μ*_1_, *μ*_2_, *π*, and *σ*. Applying the MCMC technique with our method, we can use either the posterior mean or the full posterior distribution of the bimodality index. Because the process involves estimating the distributions, the MCMC technique takes longer and is more computationally intensive. This increased time can be especially problematic for large-scale gene expression data, which makes MCMC less popular in the analysis of high-throughput genomic data. That problem, however, can be overcome by using a parallel processor system, which involves breaking the expression dataset into several small subsets and performing the analysis in parallel for each subset. In this way, the MCMC algorithm can be carried out in a relatively short time. Although a number of MCMC algorithms are available, we only applied one MCMC technique in this investigation. We chose the hybrid MCMC technique because it is not difficult to implement and was readily available. (We did not intend to evaluate which of the MCMC algorithms performed better).

One potential objection to our method is that it assumes both components are normally distributed with the same variance. Both model-based clustering via the EM algorithm and MCMC techniques can be extended to use mixtures of t-distributions^19^ or mixtures of other distributions. As long as the distributions being used have a “central parameter” that plays the role of the mean for the normal distribution, the definition of the bimodality index proposed here carries over directly to these more general mixture distributions. One can also accommodate different standard deviations by defining the standardized distance between means to be *δ* = *|μ*_1_/*σ*_1_ *– μ*_2_/*σ*_2_*|*The performances of these kinds of extensions to the method deserve further study.

We evaluated the sensitivity and specificity of the proposed method in identifying meaningful bimodal measurements through simulation studies. We compared the results of computing the bimodality index by two different algorithms, MCLUST and MCMC. We also compared the results obtained from our method with those obtained from the commonly used BIC. Due to the similarity in application between AIC and BIC, we did not include AIC in the comparison. In the null distribution, the results were quite similar; all methods had few false positives. In addition to the null distribution, we evaluated bimodal patterns with various parameter settings. Those simulations indicate that, although BIC is more sensitive for detecting bimodal distributions, using the bimodality index provides fine-grained control over the discoveries, allowing the researcher to rank the genes by their degree of bimodality and thus focus attention on the genes whose bimodal patterns are most believable and most likely to be clinically useful.

We further applied our proposed method to a dataset from a breast cancer gene expression profiling study. Unlike simulated data, actual gene expression data is far more complex and contains a high level of noise; therefore we expected a much higher rate of false discovery in that analysis, particularly when the size of one of the groups was small. When we applied the mixture-model-based clustering technique with BIC, over 35% of the genes were identified as bimodal. We tried to remove some noise prior to the analysis by setting various filtering conditions. After filtering, we still identified a substantial number of bimodal measurements when using BIC. We then applied our method to the filtered datasets. In contrast, our method produced much more reasonable results, which suggests that it is a useful approach for the analysis of real datasets. Moreover, the filtering process helped to remove some unreliable measurements for small values of *BI* (*BI* ≤ 1.3). For a strong bimodal pattern (*BI* ≥ 1.4), the results indicate that the bimodal measurements identified in this process were almost unchanged, regardless of whether or not the filtering conditions were applied. Further, the genes identified as most strongly bimodal appeared to be related to the hormone receptor (ERS1 and PGR) status and HER2 status of the breast cancer patients. Since these characteristics are known to be strong determinants of both gene expression and response to specific treatments in breast cancer, this finding provides evidence that using our method has the potential to focus on biologically important subsets of gene expression profiling data.

In addition to finding the expected genes, we described two additional genes (CKB and BST2) that exhibit strong bimodal expression patterns within breast cancer samples. Creatinine kinase, brain (CKB) is a cytoplasmic enzyme involved in energy homeostasis. It acts as a homodimer (CK-BB) in brain as well as in other tissues, and as a heterodimer with a similar muscle isozyme (CKM) in heart. Rubrey et al reported that 34% of breast cancer patients showed elevated serum levels of the CK-BB homodimer, and that an increased incidence of elevated serum CK-BB levels was associated with advanced stage disease.^20^ Using a more sensitive assay, Zarghami et al measured CK-BB levels in breast tumor cytosols and found that CK-BB was associated with more aggressive tumors but concluded that its value as a prognostic indicator was limited.^21^

Bone marrow stromal cell antigen 2 (BST2 or CD317) was originally recognized as a surface antigen on bone marrow stromal cell lines; however, it is predominantly expressed in liver, lung, heart, and placenta, and not typically expressed in normal breast. Becker et al using Affymetrix HuGeneFL and Hu95Av2 microarray experiments, identified BST2 as significantly up-regulated in a tamoxifen-resistant cell line derived from the mammary tumor cell line MaCa 3366.^22^ Recently, Cai et al reported that BST2 is up-regulated in breast cancer with bone metastasis, and concluded that BST2 may be a potential biomarker in breast cancer with bone metastasis.^23^

Our method performed reasonably well in revealing meaningful bimodal patterns of gene expression in comparison with the commonly-used BIC approach. The results from the analyses suggest that our proposed method is a sensible approach for the analysis of large-scale gene expression data, and can be extended for broad application.

## Figures and Tables

**Table 1:** Performance in simulated null distributions (*δ* = 0).

Sample sizes (n) (with 500 measurements)	Percentage of measurements identified as bimodal (false-positive rate)			
	BI cutoffs	MCMC estimated	MCLUST estimated	BIC
**50**	**1.1**	0.8	1.8	2.6
	**1.2**	0.2	1.4	
	**1.3**	0.0	1.4	
	**1.4**	0.0	1.2	
	**1.5**	0.0	0.8	
**100**	**1.1**	0.4	1.2	1.6
	**1.2**	0.2	0.8	
	**1.3**	0.2	0.8	
	**1.4**	0.0	0.4	
	**1.5**	0.0	0.0	
**200**	**1.1**	0.0	0.4	0.6
	**1.2**	0.0	0.4	
	**1.3**	0.0	0. 0	
	**1.4**	0.0	0.0	
	**1.5**	0.0	0.0	
**300**	**1.1**	0.0	0.2	0.4
	**1.2**	0.0	0. 0	
	**1.3**	0.0	0. 0	
	**1.4**	0.0	0. 0	
	**1.5**	0.0	0. 0	

**Table 2a:** Results from simulated bimodal data set, with sufficient sample sizes in each subgroup (Proportion in one group *π* = 0.3 to 0.7, in 0.1 intervals).

Sample sizes (n) (data = 500 × n)	BI cutoffs	Identified bimodal (%)											
		δ = 2			δ = 3			δ = 4			δ = 5		
		MCMC estimated	MCLUST estimated	BIC selected	MCMC estimated	MCLUST estimated	BIC selected	MCMC estimated	MCLUST estimated	BIC selected	MCMC estimated	MCLUST estimated	BIC selected
**50**	**1.1**	6.0	8.2	8.8	47.6	50.0	50.0	93.6	98.4	98.4	100	96.2	100
	**1.2**	4.0	8.8		40.2	49.8		92.0	98.4		100	96.2	
	**1.3**	2.8	7.6		33.4	49.6		89.8	98.2		100	96.0	
	**1.4**	1.4	6.8		26.6	47.4		85.8	97.6		100	95.8	
	**1.5**	0.6	5.8		17.8	43.6		78.8	95.0		100	95.6	
**100**	**1.1**	6.6	8.8	9.6	77.2	83.2	83.2	99.4	100	100	100	99.4	100
	**1.2**	3.2	8.4		67.0	82.0		98.8	100		100	99.4	
	**1.3**	1.2	6.0		53.8	76.8		98.2	99.8		100	99.4	
	**1.4**	0.4	2.8		37.8	63.4		96.6	99.0		100	99.2	
	**1.5**	0	1.4		23.2	43.2		94.2	97.6		100	99.2	
**200**	**1.1**	5.4	13.2	17.2	91.6	97.4	98.0	99.8	100	100	100	100	100
	**1.2**	2.0	9.0		83.8	95.4		99.8	100		100	100	
	**1.3**	0.4	2.0		70.6	85.4		99.8	100		100	100	
	**1.4**	0.2	0.2		48.2	66.4		99.6	99.8		100	100	
	**1.5**	0	0		23.6	40.4		99.3	99.8		100	100	
**300**	**1.1**	7.8	18.2	27.6	94.8	99.8	100	99.8	100	100	100	100	100
	**1.2**	2.6	8.2		88.6	97.0		99.8	100		100	100	
	**1.3**	0.4	1.8		73.8	85.6		99.8	100		100	100	
	**1.4**	0	0		47.8	63.6		99.8	100		100	100	
	**1.5**	0	0		22.4	35.2		99.8	100		100	100	

**Table 2b:** Results from simulated bimodal data set, with small sample size in one subgroup (Proportion in one group, *π* = 0.1, 0.2, 0.25, 0.8, 0.9).

Sample sizes (n) (data = 500 × n)	BI cutoffs	Identified bimodal (%)											
		δ = 2			δ = 3			δ = 4			δ = 5		
		MCMC estimated	MCLUST estimated	BIC selected	MCMC estimated	MCLUST estimated	BIC selected	MCMC estimated	MCLUST estimated	BIC selected	MCMC estimated	MCLUST estimated	BIC selected
**50**	**1.1**	4.8	8.0	11.0	25.2	43.6	51.4	58.2	83.0	91.6	69.4	93.6	98
	**1.2**	2.0	7.0		18.8	39.2		52.2	77.4		67.8	91.0	
	**1.3**	1.0	5.4		13.2	32.2		45.8	69.0		65.2	87.8	
	**1.4**	0.8	3.6		7.8	22.2		38.0	59.6		60.8	83.6	
	**1.5**	0.0	2.6		5.4	14.0		31.2	48.6		56.2	77.4	
**100**	**1.1**	2.8	5.8	11.0	31.8	48.6	77.2	75.8	87.8	98.4	85.6	97.2	100
	**1.2**	1.8	3.4		21.2	37.0		69.8	79.8		84.2	94.8	
	**1.3**	0.8	2.0		13.2	24.5		60.0	71.4		80.8	91.6	
	**1.4**	0.2	1.4		6.6	14.4		51.6	61.4		77.0	87.8	
	**1.5**	0	0.2		1.6	7.0		39.6	50.4		71.4	81.6	
**200**	**1.1**	0.2	3.6	23.2	41.4	53.6	95.8	80.4	90.4	100	89.9	99.6	100
	**1.2**	0	0.4		27.4	39.4		72.2	80.0		88.0	98.0	
	**1.3**	0	0		12.4	21.0		63.6	69.6		85.4	93.8	
	**1.4**	0	0		5.4	9.2		53.8	60.4		79.8	88.6	
	**1.5**	0	0		1.8	3.6		43.2	49.8		74.0	81.0	
**300**	**1.1**	0.2	2.4	35.0	46.4	55.4	99.6	83.8	91.8	100	90.8	99.8	100
	**1.2**	0	0		26.4	38.2		72.8	81.4		89.4	99.4	
	**1.3**	0	0		12.0	16.8		64.0	69.2		86.8	96.6	
	**1.4**	0	0		3.0	5.4		56.0	60.2		80.0	90.0	
	**1.5**	0	0		0.2	0.4		46.4	52.4		71.8	79.9	

**Table 3:** Identified bimodal genes from breast cancer dataset at different settings.

Quantiles set for filtering	Survived genes after filtering	Identified bimodal genes using various BI cutoffs										
		MCMC					MCLUST					
		BI = 1.1	BI = 1.2	BI = 1.3	BI = 1.4	BI = 1.5	BI = 1.1	BI = 1.2	BI = 1.3	BI = 1.4	BI = 1.5	BIC
**0.00**	22283	1238	763	463	280	181	1860	1125	675	379	213	7859
**0.25**	18570	1199	752	458	278	180	1768	1089	662	375	212	7422
**0.30**	17777	1178	740	454	278	180	1729	1074	654	374	212	7231
**0.35**	16910	1157	732	451	277	179	1684	1052	647	372	211	6974
**0.40**	16054	1131	722	448	277	179	1635	1037	641	368	211	6718
**0.45**	14996	1090	704	437	273	179	1559	993	619	360	209	6359
**0.50**	13888	1040	674	422	268	177	1465	943	599	353	205	5975

**Table 4:** Commonly identified bimodal genes using proposed method and with MCMC and MCLUST.

Quantiles set for filtering	Bimodality Index cutoffs				
	BI = 1.1	BI = 1.2	BI = 1.3	BI = 1.4	BI = 1.5
**0.00**	1113	673	397	235	151
**0.25**	1077	664	393	233	150
**0.30**	1057	653	390	233	150
**0.35**	1036	646	387	232	149
**0.40**	1012	637	384	232	149
**0.45**	967	617	372	228	149
**0.50**	919	590	361	224	147

**Table 5:** Estimated parameters for ERS, HER2 and PGR genes.

Genes	Estimated from MCMC					Estimated from MCLUST				
	μ_1_	μ_2_	σ	δ	π_1_ and π_2_	μ_1_	μ_2_	σ	δ	π_1_ and π_2_
**ERS1**	6.90	10.79	1.00	3.90	40.9% and 60.1%	6.88	10.79	0.97	4.02	38.4% and 61.6%
**HER2**	9.36	12.68	0.86	3.89	19.0% and 81.0%	9.34	12.63	0.83	3.97	21.6% and 78.4%
**PGR**	4.62	6.98	0.62	3.84	26.8% and 73.2%	4.62	6.98	0.60	3.95	26.0% and 74.0%
